# Nationwide school malaria parasitaemia survey in public primary schools, the United Republic of Tanzania

**DOI:** 10.1186/s12936-018-2601-1

**Published:** 2018-12-05

**Authors:** Frank Chacky, Manuela Runge, Susan F. Rumisha, Pendael Machafuko, Prosper Chaki, Julius J. Massaga, Ally Mohamed, Emilie Pothin, Fabrizio Molteni, Robert W. Snow, Christian Lengeler, Renata Mandike

**Affiliations:** 1 Ministry of Health, Community Development, Gender, Elderly, and Children, Dodoma, Tanzania; 2 National Malaria Control Programme, Dar es Salaam, Tanzania; 3 Swiss Tropical and Public Health Institute, Basel, Switzerland; 4 University of Basel, Basel, Switzerland; 5 National Institute for Medical Research, Dar es Salaam, Tanzania; 6 Ifakara Health Institute, Dar es Salaam, Tanzania; 7 KEMRI-Welcome Trust Research Programme, Nairobi, Kenya; 8 Centre for Tropical Medicine and Global Health, Nuffield Department of Clinical Medicine, University of Oxford, Oxford, UK

**Keywords:** Malaria, School children, Malaria surveillance, Malaria prevalence, Mosquito net use, Tanzania

## Abstract

**Background:**

A nationwide, school, malaria survey was implemented to assess the risk factors of malaria prevalence and bed net use among primary school children in mainland Tanzania. This allowed the mapping of malaria prevalence at council level and assessment of malaria risk factors among school children.

**Methods:**

A cross-sectional, school, malaria parasitaemia survey was conducted in 25 regions, 166 councils and 357 schools in three phases: **(**1) August to September 2014; (2) May 2015; and, (3) October 2015. Children were tested for malaria parasites using rapid diagnostic tests and were interviewed about household information, parents’ education, bed net indicators as well as recent history of fever. Multilevel mixed effects logistic regression models were fitted to estimate odds ratios of risk factors for malaria infection and for bed net use while adjusting for school effect.

**Results:**

In total, 49,113 children were interviewed and tested for malaria infection. The overall prevalence of malaria was 21.6%, ranging from < 0.1 to 53% among regions and from 0 to 76.4% among councils. The malaria prevalence was below 5% in 62 of the 166 councils and above 50% in 18 councils and between 5 and 50% in the other councils. The variation of malaria prevalence between schools was greatest in regions with a high mean prevalence, while the variation was marked by a few outlying schools in regions with a low mean prevalence. Overall, 70% of the children reported using mosquito nets, with the highest percentage observed among educated parents (80.7%), low land areas (82.7%) and those living in urban areas (82.2%).

**Conclusions:**

The observed prevalence among school children showed marked variation at regional and sub-regional levels across the country. Findings of this survey are useful for updating the malaria epidemiological profile and for stratification of malaria transmission by region, council and age groups, which is essential for guiding resource allocation, evaluation and prioritization of malaria interventions.

**Electronic supplementary material:**

The online version of this article (10.1186/s12936-018-2601-1) contains supplementary material, which is available to authorized users.

## Background

Tanzania is currently under epidemiological transition from meso-endemic to hypo-endemic levels characterized by marked heterogeneity across and within regions and/or councils [1]. This calls for an accurate and timely estimate of the spatial–temporal distribution of malaria transmission, malaria burden, and the impact of deployed control interventions. Since 2000 it has been estimated that the malaria burden has shown a marked decline in **s**ub-Saharan Africa, due to a large scale-up of control interventions [2, 3].

Malaria prevalence among school-aged children in Tanzania is under-researched and not well understood. To date, few data have been collected in older children in a small-scale study, which have shown an increasing proportion of the malaria burden in adolescents despite transmission falls in the general population [4, 5]. In addition, the observed high heterogeneity of malaria transmission calls for the timely identification of populations and areas at greatest need for additional interventions [6–8]. The major sources for malaria data are health management information systems (HMIS) and large household surveys, such as the malaria indicator surveys (MIS) and Tanzania Demographic and Health Surveys (TDHS). However, the HMIS captures only malaria cases for those seeking care at health facilities, while household surveys which are conducted every 4–5 years have been a useful tool to inform on the prevalence of malaria in the country. Household surveys are logistically complex, expensive, time consuming, and have limited scope of sample size, whereby in Tanzania they focus only on children under 5 years old [9]. Additionally, unsteady funding and weak health systems hinder the timely and reliable collection of data on malaria cases [6].

In this context, school surveys have gained increased attention for national surveillance, complementing household surveys [10–12]. Schools are often well organized and easily accessible and provide the possibility of collecting malariometric and control data from many children in a short period of time and at a low cost compared to TDHS/MIS, which are too costly for routine surveillance. The importance of school survey data for the planning of targeted interventions was demonstrated by school surveys conducted in other African countries, such as Kenya, Ethiopia, Uganda, Malawi, Côte d’Ivoire, Democratic Republic of Congo, and The Gambia [10–21], summarized by Brooker et al. [9].

A national, school, malaria parasitaemia survey (SMPS) was conducted to close that data gap by increasing the scope from children under 5 years to children 5–16 years old and increase and complement the power of the population surveys through increased sites and sample size. The SMPS was designed to allow estimates of malaria prevalence and determine spatial and temporal risks of *Plasmodium falciparum* transmission among public primary school-age pupils in mainland Tanzania. The objectives were: (1) to determine the prevalence of malaria among public primary school-enrolled pupils; (2) to establish the spatial and temporal risks of *P. falciparum* transmission across malaria-endemic councils; and, (3) to determine the access and use of insecticide-treated bed nets among school-age children.

## Methods

### Malaria in Tanzania

Around 95% of the population is at risk of malaria [22]. Malaria transmission has been described as unstable, seasonal in the arid central plateau, as stable seasonally in the southern part (unimodal) and northern and western parts (bimodal), and stable throughout the year in the coastal fringe, southern lowlands and the Lake Zone [1]. In the past decade, there was an overall decrease in malaria prevalence among children under the age of 5 years from 18.9 to 9.5% between 2008 and 2012 [23, 24]. However, the decline was not everywhere and even increased in some parts of the country, leading to an overall prevalence of 14% in 2016, observed in the 2015–2016 TDHS-MIS survey [25].

### Geography and population

Tanzania is among the East African countries, with a total area of 945,000 sq. km. Mainland Tanzania is divided into regions, councils, wards, and villages. Tanzania is mostly rural, with around 28% of the population living in urban councils, while 10% of the population live in Tanzania’s largest city Dar es Salaam. According to the 2012 population census, Tanzania has almost 45 million inhabitants, with half of the population younger than 17 years [26].

### Climate and seasonality

The climate in Tanzania is tropical with temperatures between 25 and 31 °C during the hottest period (November to February) and between 15 and 20 °C in the cooler period (May to August). In most parts of the country, the temperature rarely falls below 20 °C, while in highland areas the temperature ranges from 10 to 20 °C throughout the year. There are two different rainfall seasons in Tanzania: (1) from December to April in southern, southwestern, central and western parts of Tanzania, and (2) from October to December and March to May in northern and northern coastal parts of Tanzania [27].

### Education system and schools

In Tanzania, in the year 2002, primary education had been made compulsory to all eligible children [28]. According to the Ministry of Education and Vocational Training data in 2015, there were 16,960 registered primary schools in mainland Tanzania, whereas 928 (5.5%) of those were non-governmental [29]. The school enrolment is described through intake and enrolment ratios, whereas the National Intake Ratio (NIR) is the percentage of new enrolments of children of official school entrance age over all children of official school entrance age [30]. In 2014 it was estimated that the net enrolment ratio ranged from 73% in Katavi region to nearly 100% in Iringa region [30].

### School malaria parasitaemia survey (SMPS)

#### Survey design, procedures and study tools

**Fig. 1:**
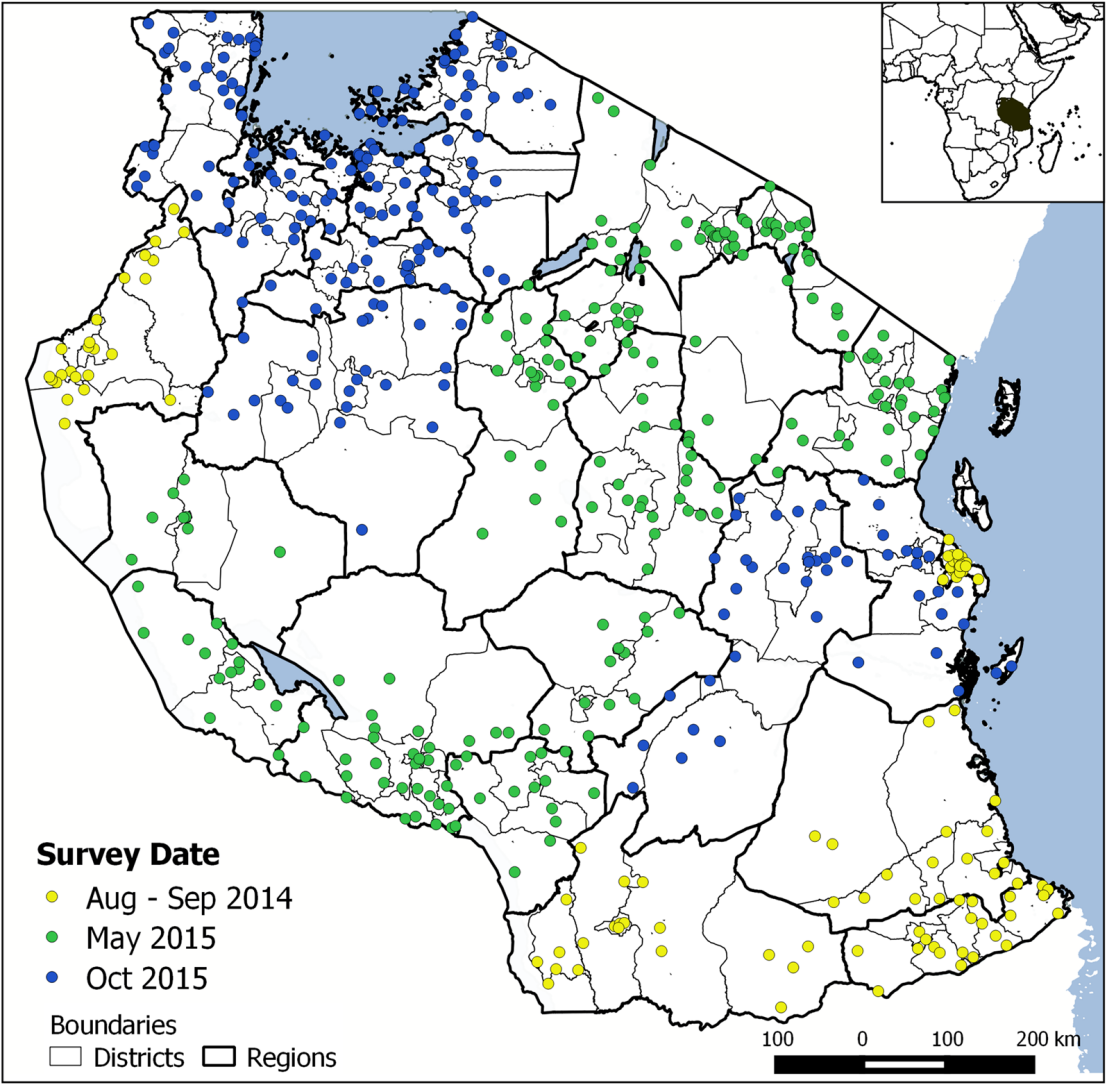
Locations of sampled schools (N = 537) coloured by survey date

A cross-sectional, school, malaria parasitaemia survey was conducted in three phases: phase I which was considered a pilot phase, included 113 schools in 5 regions between August and September 2014; phase II included 217 schools in 11 regions in May 2015; and, phase III included 207 schools in 9 regions in October 2015 (Fig. 1). The sample was calculated and selected using a multistage, stratified, proportional probability sampling method. In each council, the number of children to be tested was calculated based on the known population figures [26] and estimated mean population-weighted parasite prevalence rate adjusted for children aged 2–10 years (*PfPR*_2–10_) for 2010 [1]. The number of children to be tested was then used to determine the number of schools included in the survey in each council, assuming an average of 100 children per school. As a next step, the list of wards in each council was confirmed and updated during a national orientation meeting by local expert teams from health and education sectors. The councils were stratified according to the altitude, population density, demographic characteristics, and any other features available, such as urban/rural. The number of strata was equal to the pre-defined number of schools, aiming to select one school within each stratum. The stratification was done to ensure that the study design captured the heterogeneity of malaria transmission at sub-council level. In each stratum one ward and subsequently one village were randomly selected, using a list of the administrative units obtained from the National Bureau of Statistics Tanzania [31]. Thus, the probability of a school to be selected was dependent on the number of wards within the strata and the number of villages within the selected ward. In case there was more than one school located in a village, the school was randomly selected. Proportionate stratification was used to obtain the actual number of children to be tested at each school. In each school, all seven primary school classes were included with an equal number of children in each class, balancing the sample for gender groups. In phase III, only classes one to six were included, due to the near end of the school term for children in class seven. The selection of children in each class was done separately for boys and girls, using a systematic sampling procedure from a class register. A detailed flowchart about the sampling design can be found in Additional file 1: Fig. S1.

An average of 8 days was used for data collection per region, ranging from 4 to 15 days. Within councils the data collection took on average 4 days, ranging from 1 to 11 days (Additional file 2: Fig. S2). In each surveyed school, a total of 2 days was used: one day for planning, sampling and consenting and a second day for interviewing and testing. Each council was given an additional 1 day for data management and reporting. To test the children for malaria parasites, SD BIOLINE Malaria Ag P.f/Pan rapid malaria tests were used (Standard Diagnostics Inc., Republic of Korea). The individual test results were recorded in the designated RDT register and in the respective individual children’s questionnaire. Furthermore, the questionnaire captured information on household size, bed net ownership and use, school absenteeism and fevers during the 2 weeks prior the survey and, reported education level of one parent was obtained in phase II and III. Children with a positive test result were treated with artemether–lumefantrine (ALu), as recommended in the National Malaria Diagnosis and Treatment Guidelines [32] and referred to the nearest health facility when necessary. To confirm the consistency of recording of malaria test results, 20% of the used rapid diagnostic tests were reviewed by quality assurance team and the results were compared with the recorded test results on the paper forms. For wrongly recorded test results, the amendment was handled at the study site by drawing a single line through an incorrect value and dated.

#### Survey staff and training

Data collection was carried out by council teams, including the malaria focal person, two laboratory technicians, one education officer, and one teacher from each surveyed school. Regional teams, including the regional malaria focal person and a national supervisor from the National Malaria Control Programme (NMCP), National Institute for Medical Research (NIMR) or Ifakara Health Institute (IHI), supervised data collection at regional level and visited one council team per day to assess the performance of malaria testing, assess data quality issues and individuals’ interview. All teams participated in a national 3-day training directly before the start of the data collection.

### Ethical considerations

Ethical clearance was given by the National Health Research Ethics Committee of NIMR. Informed consent for the survey was based on a passive, opt-out method of parental permission. Schools were instructed to inform the students and parents concerning the survey. It was assumed that parents approved their children’s participation if they did not express their disapproval.

### Data entry

Paper forms and RDT kits were transported to the head office of the NMCP in Dar es Salaam for data entry and storage. Data were single-entered by a group of trained data entry clerks, using EpiData (EpiData Association, Denmark) templates in phases I and III, and Microsoft Excel (Microsoft Corporation, Seattle, USA) in phase II, under supervision of study investigators and a statistician. Entered data into Epi-data were exported into Excel for daily simple quality checks. Daily quality checks were performed by comparing entered data per data entry clerk for data entry errors using the filter function in Excel. After data entry, 20% of data from each data entry clerk were validated by comparing entered data with paper forms. The whole dataset was screened for suspicious data entries such as invalid values, out of range or missing values, following a checklist for each variable. Suspicious entries were recorded, and hard copies revisited to diagnose the entries as erroneous with correction, or as invalid.

### Data analysis

The data were analysed in STATA version 14 [33] and maps were created using QGIS **[**34**]**. Descriptive statistics were done for all variables. Prevalence and proportions, together with their 95% confidence intervals, were calculated, adjusted for clustering effect of children between schools. Chi square tests were used to compare characteristics of children and schools with the outcome variables “malaria infection” and “bed net use”.

### Variable definitions and sources

The age range was categorized into three groups: 5 to under 9 years, 9 to 12 years, and older than 12 years. Councils were classified as urban or rural based on the type of council. Municipals, township authorities and city councils were classified as urban, remaining councils were classified as rural. Transmission zones were classified according to the categories used in the most recent epidemiological profile and based on the predicted mean *PfPR*_2–10_ from 2010, adjusted for the ages 2–10 years [1]. The following categories were created: low stable (*PfPR*_2–10_ < 1%), hypo-endemic 1 (*PfPR*_2–10_ 1 to < 5%), hypo-endemic 2 (*PfPR*_2–10_ 5 to < 10%), meso-endemic (*PfPR*_2–10_ 10 to < 50%), and hyper-holo-endemic (*PfPR*_2–10_ ≥ 50%). Bed net ownership was defined as having at least one mosquito bed net in a household. Values presented on malaria infection type [e.g. *P. falciparum*, other *Plasmodium* species or Pf and pan infection (when control and other two lines appear)], were based on the RDT designated register dataset (N = 49,169), which included slightly more children than the questionnaire dataset (N = 49,113). The altitude was extracted for the geo-location of the schools, using data from Shuttle Radar Topography Mission [35], downloaded from WorldClim [36, 37]. The altitude was categorized into: below 750 m, 750–1250 m, 1250–1750 m, and above 1750 m. Further environmental variables added were: ecozone and temperature suitability index (TSI). The ecozones were classified by the Food and Agriculture Organization (FAO) as tropical rain forest, tropical moist deciduous forest, tropical dry forest, tropical shrubland, and tropical mountain system [38]. The temperature suitability index is a relative measure of the impact of the temperature on vectorial capacity (number of infectious mosquitoes), ranging from 0 to 1 [39]. The TSI raster file for 2015 was downloaded from the Malaria Atlas Project at the University of Oxford [40] and mean values were extracted per council using R version 3.3.1 [41].

Population data were obtained from the national population census 2012, available from the National Bureau of Statistics (NBS) Tanzania [42], and gridded population densities from WorldPop [43]. Bed net use for a child was defined as a binary variable stated as “yes” if a child reported general use of net regardless of bed net ownership and “no” otherwise. Malaria infection was defined as “yes” for a positive RDT case regardless of the malaria infection type and “no” if no infection was detected.

### Multilevel mixed-effects logistic regression analysis

To assess the influence of risk factors for malaria infection and bed net use, multilevel mixed-effects logistic regression models were fitted for each outcome separately. Data were assumed to be clustered at council and schools; hence, these were included in the model as random effects. Variable selection was based on available environmental, socio-economic and individual covariates, which were previously associated with malaria, such as annual rainfall, temperature and vegetation index, TSI, altitude, type of council, reported education of parents, gender, age, fever in the 2 weeks prior to survey. In addition, log-likelihood ratio tests of bivariate models, with a cut-off at a significance level of 0.01 were used. In the model with outcome “malaria infection” the following interaction terms were assessed: gender and bed net use, age and bed net use, reported parental education level and bed net use, reported parental education level and urban area. Interaction terms were included at a significance level of 0.05. The final set of selected variables, which was used in both models included: gender, age, bed net use or RDT result, reported parental education, type of council, school-point altitude, geographical zone, and school-point TSI. Due to limited available variables in phase I, two multivariable models were calculated: model I included data from all regions but had fewer covariates (N = 47,157; 96%), whereas model II excluded data from phase I regions but more covariates (N = 30,715; 62.5%) (Table 1).

**Table 1:** Multivariable regression models used in the present analysis

	Model I	Model II
Regions	All	Excluding phase I data: Kigoma, Mtwara, Lindi, Ruvuma, Dar es Salaam
Number of observations	47,157 (96%)	30,715 (62.5%)
Variables		
Included	Gender, age, bed net use or RDT result, type of council, altitude, geographic zone, TSI	Gender, age, reported parental education, bed net use or RDT result, type of council, altitude, eco-zone, geographic zone, TSI
Excluded	Reported parental education, eco-zone	
Random effects	Council, school	Council, school

## Results

Overall 49,113 children in 537 schools were tested for malaria and interviewed. On average 91 children were sampled in each school, ranging from 44 to 199 children. Regarding the transmission zones, 7606 (15.5%) children were sampled in councils classified as low stable, 11,845 (24.1%) in hypo-endemic 1; 8188 (16.7%) in hypo-endemic 2; 21,208 (43.3%) in meso-endemic; and, 146 (0.3%) in hyper-holo-endemic (Table 2).

**Table 2:** Number of included councils, schools and children, by transmission area

	Councils				Schools				Children		Total pop. (2010) *, %
	N	Children per council			N	Children per school			N	%	
		Mean	Min	Max	%	Mean	Min	Max			
Transmission zone*											
Low stable	30	253.5	111	546	88	86.4	44	166	7606	15.5	25.4
Hypo-endemic 1	40	296.1	64	501	130	91.1	42	157	11,845	24.1	20.4
Hypo-endemic 2	27	303.3	97	653	91	90	44	199	8188	16.7	13.3
Meso-endemic	67	316.5	89	1109	224	94.7	44	165	21,208	43.2	38.4
Hyper-holo-endemic	1	146	146	146	2	73	73	73	146	0.3	2.3
Missing**	1	120	120	120	2	60	60	60	120	0.2	–
Total	166	295.9	64	1109	537	91.5	42	199	49,113	100	100

* Source: Epidemiological profile Tanzania, 2013 [1]; ** no data for Mafia council in Pwani Region

### Characteristics of sample

Malaria test results were available for 49,102 (99.9%) children and bed net use information was available for 47,800 children (97.3%). Majority of the children were sampled in rural councils (80%). The mean altitude was 1016 m (inter-quartile-range 615–1351 m) and the mean TSI was 0.4 (inter-quartile-range 0.22–0.57). The sample included as many boys as girls (boys 49.2%), and the children were on average 11 years old (range 4–20; inter-quartile-range 9–13 years) (Additional file 3: Fig. S3). Girls were found to be on average one year younger than the boys. Majority of the children reported having parents with primary school education (N = 23,445; 72%), while around 10% of the children reported having parents who had never been to school (N = 3736; 11.6%). In the 2 weeks prior to the survey, 17,272 (35.7%) children were absent due to sickness and 15,709 (32.7%) had a fever; of those, 8383 (53.4%) children went to a health facility for treatment and reported to be diagnosed with malaria. Almost all the children diagnosed with malaria had received treatment (N = 8155, 97.7%), while 1421 (14.8%) children received treatment but were not diagnosed with malaria. In total, 41,914 (89.5%) children reported having at least one bed net at home, while the bed net use was 69.6%. In urban councils significantly, more children reported to sleep under a bed net than in rural councils (82.20%, 95% CI 78.7–85.2% vs 66.50%, 95% CI 64.1–68.8%) (Table 3).

**Table 3:** Sample characteristics and risk factors for malaria infection and bed net use in school children in Tanzania

	Total children		Malaria (N = 49,102)			Bed net use (N = 47,800)		
			Children tested positive			Children sleeping under bed net		
	N	%	n	%	95% CI	n	%	95% CI
Total	49,113	100	10,627	22	(19.6–23.9)	33,284	70	(67.6–71.6)
Gender								
Male	24,205	49.3	5606	23.2	(21.0–25.5)	16,077	68.3	(66.1–70.3)
Female	24,681	50.3	4940	20.0	(18.0–22.2)	17,078	71.0	(68.9–73)
Missing	227	0.5	81	35.7	–	129	63.9	–
Age								
< 9	9075	18.5	1651	18.2	(16.1–20.5)	5974	68.8	(66.3–71.2)
9–12	18,892	38.5	4045	21.4	(19.2–23.8)	13,106	71.2	(69.2–73.3)
> 12	20,730	42.2	4847	23.4	(21.2–25.7)	13,957	68.6	(66.3–70.8)
Missing	416	0.9	84	20.2	–	247	62.7	–
Parental education								
No school	3736	7.6	1074	28.8	(24.4–33.5)	1930	54.7	(50.2–59.1)
Primary	23,445	47.7	5163	22.0	(19.4–24.8)	14,968	65.8	(63.1–68.3)
Secondary	4547	9.3	808	17.8	(15.1–20.9)	3430	77.5	(74.8–80)
Diploma or higher	525	1.1	52	9.9	(7.0–13.9)	415	80.7	(76.1–84.7)
Missing	5424	11.0	970	17.9	–	3617	69.7	–
Not interviewed*	11,436	23.3	2560	22.4	–	8924	78.4	–
Bed net use								
No	14,516	29.6	2943	20.3	(17.8–23.0)	–	–	–
Yes	33,284	67.8	7270	21.9	(19.6–24.2)	–	–	–
Missing	1313	2.7	414	31.5	–	–	–	–
Zone								
Eastern	6967	14.2	1269	18.2	(13.3–24.4)	5819	86.6	(83.4–89.3)
Western	4805	9.8	1449	30.2	(25.5–35.2)	3221	68.8	(64–73.2)
Southern	4002	8.1	1344	33.6	(27.2–40.8)	3255	81.6	(76.8–85.6)
Southern highlands	4116	8.4	495	12.0	(7.2–19.4)	2350	58.7	(52.4–64.7)
Southwest highlands	4867	9.9	846	17.4	(11.6–25.2)	2946	61.6	(55.3–67.5)
Central	5653	11.5	156	2.8	(1.6–4.6)	3077	55.1	(49.6–60.5)
Northern	6191	12.6	317	5.1	(3.1–8.4)	3360	55.6	(49.4–61.7)
Lake	12,512	25.5	4751	38.0	(33.6–42.5)	9256	77.2	(73.1–80.8)
Missing	0	0.0	0	0.0	–	0	0.0	–
Area								
Urban	9708	19.8	588	6.1	(4.0–9.1)	7866	82.2	(78.7–85.2)
Rural	39,405	80.2	10,039	25.5	(23.1–28.0)	25,418	66.5	(64.1–68.8)
Missing	0	0.0	0	0.0	–	0	0.0	–
Eco-zone (tropical) *								
Dry forest	7124	19.0	2247	31.5	(25.9–37.8)	5417	79.4	(74.7–83.4)
Moist decid. forest	5819	15.5	1816	31.2	(25.1–38)	3786	67.8	(62–73.1)
Mountain system	6053	16.1	723	11.9	(7.9–17.6)	3329	57.0	(50.3–63.5)
Rainforest	3331	8.9	1272	38.2	(28.6–48.7)	2664	82.8	(76.3–87.8)
Scrubland	15,216	40.5	2009	13.2	(10.3–16.7)	9078	61.2	(57.4–64.9)
Missing	11,570	23.6	2560	22.2	–	9010	77.9	–
Altitude (m)								
< 750	13,228	26.9	3248	24.6	(20.7–28.9)	10,697	82.7	(80.1–85)
750–1250	18,901	38.5	5040	26.7	(23.3–30.4)	13,284	72.0	(68.8–75)
1250–1750	14,581	29.7	2335	16.0	(12.6–20.1)	8434	59.8	(56–63.4)
> 1750	2403	4.9	4	0.2	(0.1–0.5)	869	37.8	(30.5–45.7)
Missing	0	0.0	0	0.0	–	0	0.0	–

* No data for children sampled in phase I

### Malaria infections

**Fig. 2:**
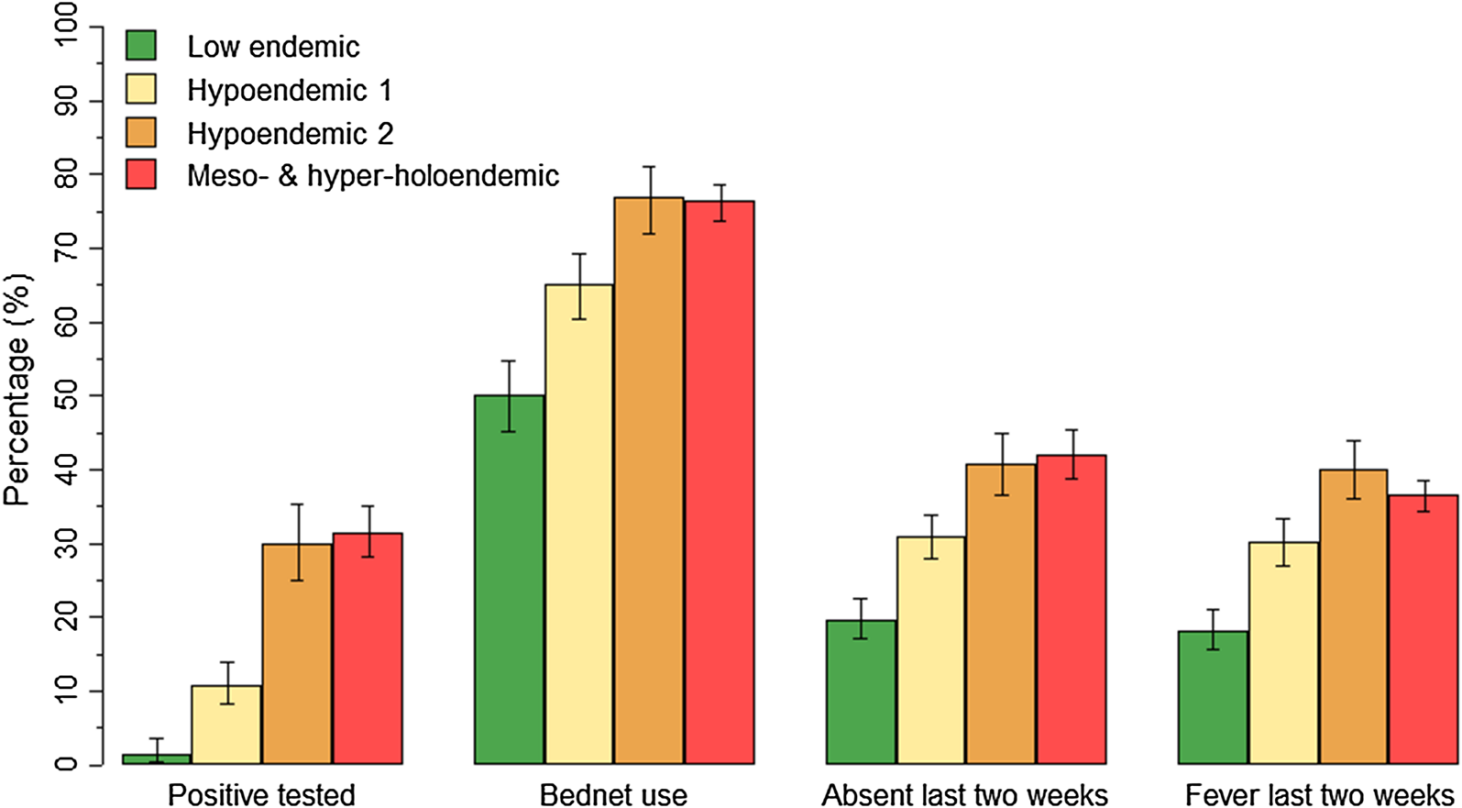
Malaria prevalence, bed net use, school absenteeism, and recent fever of school children compared by transmission zone. Error bars indicate 95% CI adjusted for school clustering

In total, 10,627 children (21.6%) tested positive for malaria (95% CI 19.5–23.9) (Table 3). Out of those, 6840 children (65%) had a mono-infection with *P. falciparum*, 3582 children (34.1%) had a malaria infection with *P. falciparum* and a non-*falciparum* parasite (*Plasmodium vivax, Plasmodium ovale* or *Plasmodium malariae*) infection and 93 children (0.9%) were only infected with a non-*falciparum* parasite. Furthermore, from the positive-tested children, 2316 children (21.8%) reported to have been diagnosed with malaria in the 2 weeks preceding the survey, and 2153 children (20.3%) received treatment. Slightly more boys than girls were tested positive [N = 5606 (23%, 95% CI 21–25.5%) vs N = 4940 (20%, 95% CI 18–22.2%)]. The malaria prevalence was 18.2% in children 5 to < 9 years (95% CI 16.1–20.5%), 21.4% in children aged 9–12 years (95% CI 19.2–23.8%) and 23.3% in children older than 12 years (95% CI 21.2–25.7%). The malaria prevalence was highest in children reported parents with no school education (28.8%, 95% CI 24.4–33.5%), compared to children with reported parents with primary (22.0%, 95% CI 19.4–24.8%), reported parents with secondary (17.8%, 95% CI 15.1–20.9%), and reported parents to have higher education (9.9%, 95% CI 7.0–13.9%). In meso-endemic, and hyper-holo-endemic councils almost one-third of the children were tested malaria positive (31.4%, 95% CI 28.1–34.9%, and 29.9%, 95% CI 25.1–35.3%, respectively), in hypo-endemic councils only 10% were tested positive (10.9%, 95% CI 8.3–14.1%), and in low stable endemic councils fewer than 2% were tested positive (1.4%, 95% CI 0.5–3.7%) (Fig. 2). The malaria prevalence slightly increased by age in all transmission zones (Additional file 4: Fig. S4). The malaria prevalence was highest at altitudes below 750 m and between 750 and 1250 m above sea level (24.6 and 26.7%) and lowest in areas 1750 m above sea level (0.2%), (16.0% at 1250–1750 m). In rural councils, the malaria prevalence was four times higher than in urban councils (25.5%, 95% CI 23.1–28.0% vs 6.1%, 95% CI 4.0–9.1%) (Table 3). Exceptions were urban councils of Geita TC, Handeni TC, Kigoma MC and Masasi TC, with prevalence higher than the national average.

**Fig. 3:**
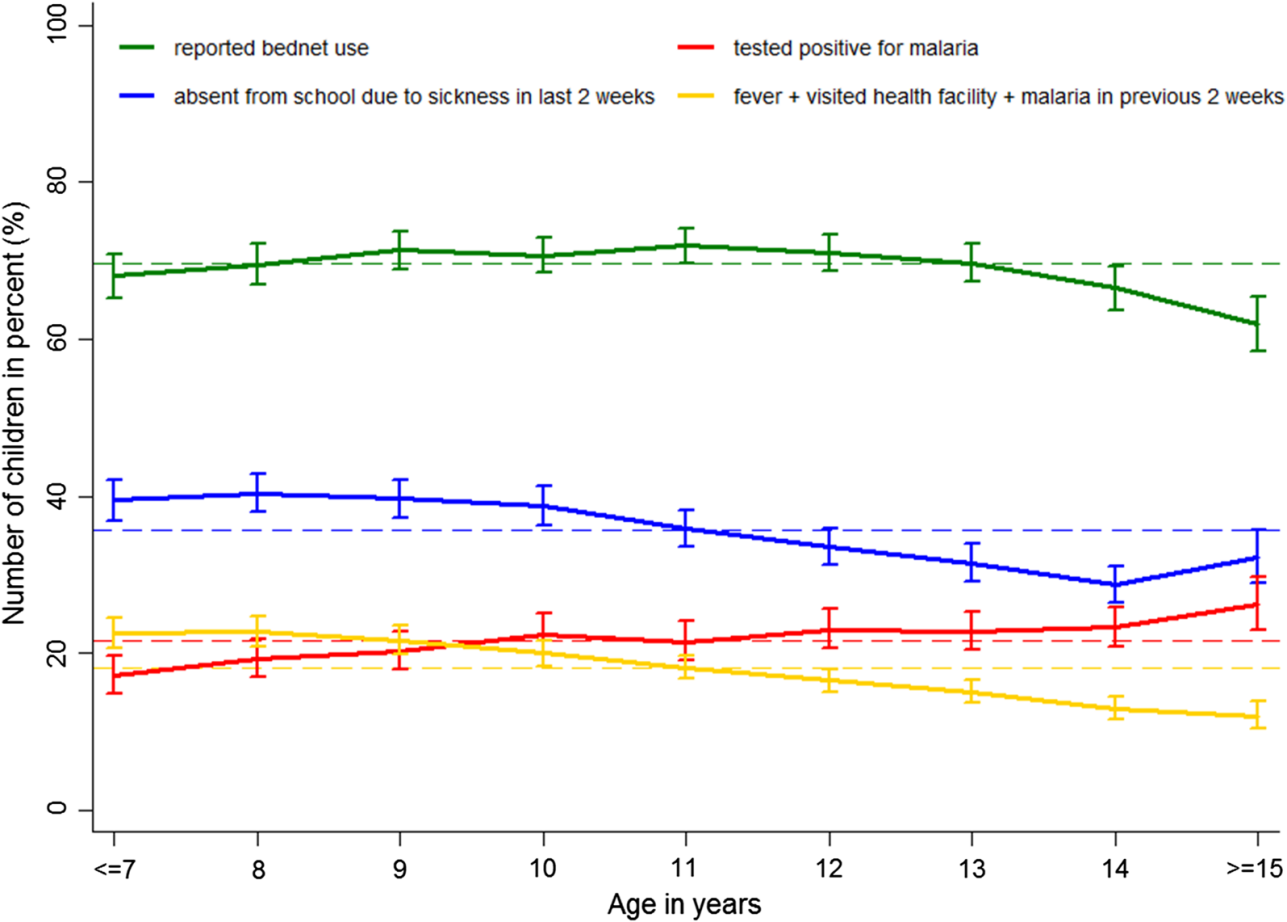
Bed net use, measured malaria prevalence, school absenteeism, and history of sickness in previous 2 weeks, by age. The error bars present the 95% CI adjusted for school clustering and the dashed line presents the mean for all ages

Figure 3 shows the main indicators among school children by age. Bed net use was high and close to the mean for all ages until it declined at age 14 years. The percentage of children who reported to be absent from school due to sickness in the 2 weeks preceding the survey was higher in younger children and decreased after the age of 11 years. The percentage of children who reported to have had a fever went to a health facility and were diagnosed with malaria was higher in younger children and started to decline after the age of 10 years. With increasing age fewer children were diagnosed with malaria at a health facility in the 2 weeks before the survey, while the percentage of children tested positive for malaria at the day of the survey showed an increasing trend with age (Fig. 3).

### Malaria infection, bed net use and associated risk factors

In the multivariable analysis^1^ girls had lower odds of malaria infection than boys (OR 0.74; 95% CI 0.69–0.79) and higher odds of sleeping under a bed net (OR 1.21; 95% CI 1.14–1.28). The association between malaria infection and age was not significant, (children 9–12 years OR 1.03; 95% CI 0.92–1.15, and children older than 12 years OR 1.05; 95% CI 0.95–1.17 compared to children 5–9 years). The association between age and bed net use was significant, but the difference in the odds ratio was only marginal (OR 1.10 vs OR 1.12). Children who reported to sleep under a bed net were less likely to have malaria (OR 0.81; 95% CI 0.74–0.88) (Table 4).

**Table 4:** Multivariable analysis of the risk factors for malaria and bed net use in primary school children in Tanzania

Outcome/covariates	Univariable		Model I (N = 47,157)		Model II (N = 30,715)			
	Malaria		Malaria		Malaria		Bed net use	
	OR (95% CI)	p- value	OR (95% CI)	p-value	OR (95% CI)	p-value	OR (95% CI)	p-value
Gender								
Male vs female	0.75 (0.71–0.79)	< 0.01	0.77 (0.72–0.81)	< 0.01	0.74 (0.69–0.79)	< 0.01	1.21 (1.14–1.28)	< 0.01
Age (years)								
< 9 vs 10–12	1.06 (0.98–1.15)	0.11	1.05 (0.96–1.14)	0.36	1.03 (0.92–1.15)	0.55	1.1 (1.01–1.20)	0.02
< 9 vs > 12	1.09 (1.01–1.18)		1.06 (0.98–1.15)		1.05 (0.95–1.17)		1.12 (1.03–1.22)	
Parental education								
Primary vs no school	1.18 (1.06–1.31)	< 0.01	–		1.18 (1.06–1.32)	< 0.01	0.69 (0.63–0.76)	< 0.01
Primary vs secondary or higher	0.74 (0.67–0.82)		–		0.75 (0.67–0.83)		1.48 (1.35–1.61)	
Bed net use								
No vs yes	0.76 (0.71–0.81)	< 0.01	0.76 (0.71–0.82)	< 0.01	0.81 (0.74–0.88)	< 0.01	–	
Malaria								
Negative vs positive	–		–		–		0.81 (0.74–0.88)	< 0.01
Area								
Rural vs urban	0.14 (0.05–0.38)	< 0.01	0.12 (0.066–0.21)	< 0.01	0.15 (0.072–0.29)	< 0.01	1.92 (1.25–2.94)	< 0.01
Altitude (m)								
< 750 vs 750–1250	0.25 (0.12–0.48)	< 0.01	0.18 (0.081–0.39)	< 0.01	0.26 (0.11–0.62)	0.02	0.43 (0.25–0.74)	0.01
< 750 vs 1250–1750	0.07 (0.04–0.15)		0.12 (0.045–0.31)		0.25 (0.088–0.72)		0.27 (0.14–0.53)	
< 750 vs > 1750	0.002 (0.00–0.02)		0.02 (0.003–0.14)		0.07 (0.0093–0.47)		0.12 (0.049–0.31)	
Temperature suitability index								
TSI (2er intervals)	3.51 (2.80–4.39)	< 0.01	–	< 0.01	2.2 (1.56–3.09)	< 0.01	1 (0.81–1.24)	0.98
Zone								
Central vs eastern	28.15 (7.68–103.20)	< 0.01	1.86 (0.60–5.72)	< 0.01	2.11 (0.63–7.01)	< 0.01	2.13 (0.96–4.71)	< 0.01
Central vs western	59.21 (15.40–228.10)		31.13 (11.6–83.4)		11.21 (3.23–39.0)		2.18 (0.91–5.19)	
Central vs southern	72.58 (17.70–296.90)		2.79 (0.83–9.33)					
Central vs southern highlands	2.13 (0.53–8.53)		2.64 (0.91–7.67)		0.41 (0.092–1.85)		0.71 (0.33–1.52)	
Central vs southwest highlands	10.33 (2.82–37.80)		6.7 (2.52–17.9)		3.19 (1.06–9.60)		1.57 (0.78–3.15)	
Central vs northern	1.05 (0.29–3.77)		0.47 (0.17–1.31)		0.43 (0.15–1.19)		0.72 (0.40–1.30)	
Central vs lake	69.76 (23.0–211.9)		40.97 (18.0–93.4)		24.43 (10.4–57.1)		3.54 (2.03–6.18)	
Eco-zone (tropical)								
Scrubland vs dry forest	4.92 (2.44–9.94)	< 0.01	–		2.06 (1.14–3.72)	0.22	0.93 (0.61–1.41)	0.81
Scrubland vs moist deci. forest	4.98 (2.31–10.73)		–		2.36 (1.21–4.60)		0.89 (0.57–1.38)	
Scrubland vs mountain system	0.56 (0.23–1.34)		–		1.51 (0.68–3.38)		1.01 (0.64–1.60)	
Scrubland vs rainforest	7.63 (2.90–20.08)		–		3.01 (1.41–6.40)		1.33 (0.76–2.31)	

### Reported education of parents

The reported education of the parents was strongly associated with both malaria infection and bed net use, with lower odds of malaria infection and higher odds of bed net use in children associated with higher educated parents. For malaria infection, the odds ratio in children with parents with no school education was 1.18 (95% CI 1.06–1.32), and in children with parents with secondary or higher education 0.75 (95% CI 0.67–0.83), compared to children with parents with primary education. The odds ratios of bed net use increased with increasing education of the parents. Children with non-educated parents had an odds ratio of 0.69 (95% CI 0.63–0.76), with secondary or higher education was 1.48 (95% CI 1.35–1.61), compared to children reported to have parents with primary education.

### Geographical zone and altitude

In comparison to children living in the Central Zone, children living in Northern and Southern Highlands had lower odds of malaria infection, and children living in the other zones had higher odds of malaria infection. For instance, children living in the Lake Zone had 24.4 times higher odds of malaria infection compared to children living in the Central Zone (95% CI 10.4–57.1). Children living in Southern Highlands and Northern Zones, were less likely to sleep under a bed net (OR 1.57; 95% CI 0.78–3.15, and OR 0.72; 95% CI 0.40–1.30), while higher odds ratios were found in children living in the Lake Zone (OR 3.54; 95% CI 2.03–6.18), compared to children living in the Central Zone. The odds of malaria infection significantly decreased at higher altitudes, with an odds ratio of 0.26 at 750–1250 m (95% CI 0.11–0.62), an odds ratio of 0.25 at 1250–1750 m (95% CI 0.09–0.72) and an odds ratio of 0.07 at altitudes higher than 1750 m (95% CI 0.01–0.47), compared to areas below 750 m. The odds for children to sleep under a bed net decreased significantly with increasing altitude (OR 0.43; 95% CI 0.25–0.74 at 750–1250 m, OR 0.27; 95% CI 0.14–0.53 at 1250–1750 m, and OR 0.12; 95% CI 0.05–0.31 at 1750 m and higher, compared to 750 m and lower).

### Council type

Living in urban councils was significantly associated with lower odds of malaria infection, compared to living in rural councils (OR 0.15, 95% CI 0.08**–**0.29). Children living in urban councils were two times more likely to sleep under a bed net than children living in rural councils (OR 1.92; 95% CI 1.25**–**2.94).

### Eco-zone

For the risk of malaria infection as well as bed net use, it made no significant difference in which eco-zone the children were living, the chance of malaria infection was higher in dry forest (OR 2.06, 95% CI 1.42–3.72), moist deciduous forest areas (OR 2.36, 95% CI 1.21–4.60), mountain system (OR 1.51, 95% CI 0.68–3.38), and three times higher in rainforest areas (OR 3.01, 95% CI 1.41–6.40) compared to scrubland areas. Regarding bed net use, children in tropical rainforest areas were more likely to use a bed net (OR 1.33; 95% CI 0.76–2.31), while children in moist deciduous forest were least likely to use a bed net (OR 0.89; 95% CI 0.57–1.38), in comparison to tropical scrubland areas (Table 4).

### Geographical distribution of observed malaria prevalence

**Fig. 4:**
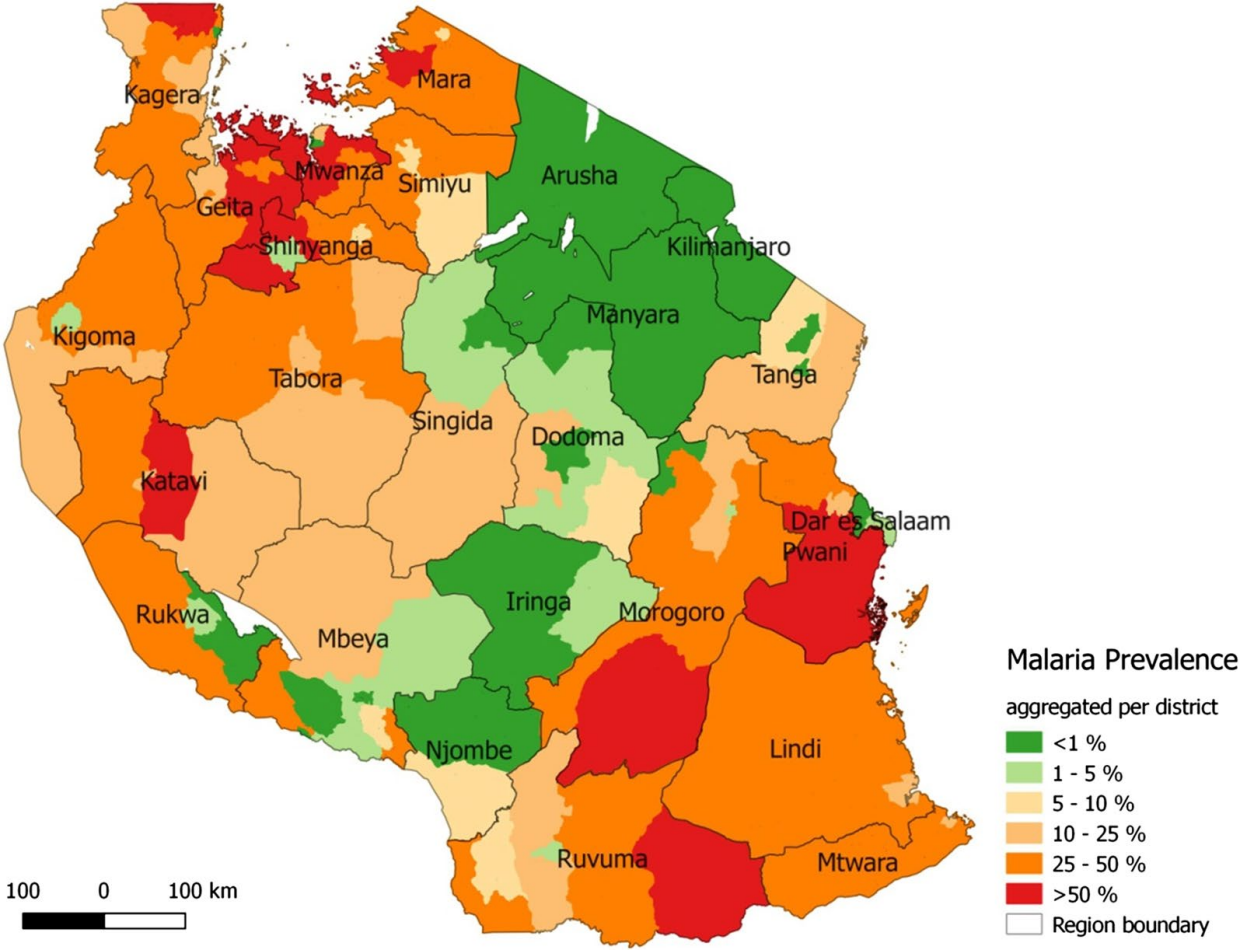
Geographical distribution of the observed mean malaria prevalence among school children per council. The prevalence shown is the unadjusted observed prevalence, measured in different times of the year

The malaria prevalence was highest in the Lake Zone (38%, 95% CI 33.5**–**42.4), Southern Zone (33%, 95% CI 26.8**–**40.5%), as well as Western Zone (30%, 95% CI 25.3**–**35%), and lowest in Central (2.8%, 95% CI 1.4**–**4.2%), and Northern zones (5.1%, 95% CI 2.5**–**7.7%). At council level, the malaria prevalence ranged from 0 to 76.4%. In 62 of the 166 councils, the malaria prevalence was below 5%, in 27 between 5 and 10%, in 25 between 10 and 25%, in 51 between 25 and 50%, and in 18 councils above 50%. Highest malaria prevalence was found in Geita, Pwani, Mwanza, and Katavi (≥ 40%); lowest prevalence in Arusha, Kilimanjaro, Manyara and Iringa (< 1%) (Fig. 4).

**Fig. 5:**
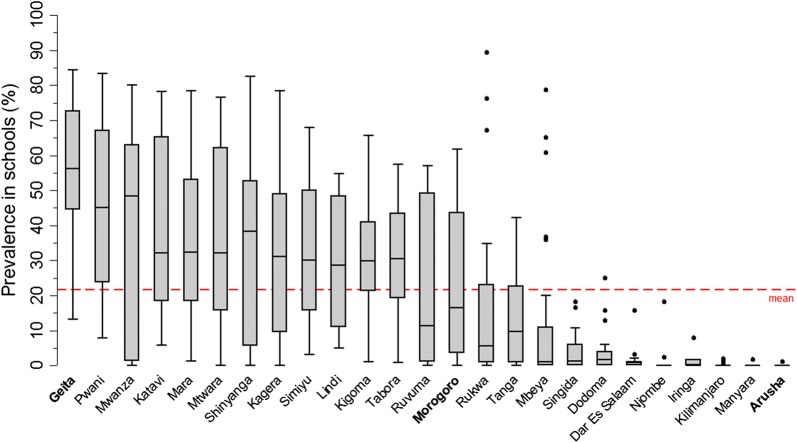
Distribution of malaria prevalence in schools by region. The regions are sorted by regional mean malaria prevalence. Geita had the highest prevalence (53.7%) and Arusha the lowest prevalence (< 0.1%). The grey box visualizes the interquartile range (25–75%) of the school prevalence within each region. The horizontal line within the grey box is the median of the school prevalence distribution. The spikes mark the lowest and highest quartile. The points present outlying schools with prevalence higher or lower most of the rest of the prevalence (1.5 times the interquartile range above the upper quartile/below the lower quartile)

Between schools, the variation of the malaria prevalence was greatest in regions with a high mean prevalence, while the variation was marked by only a few outlying schools in regions with a low mean prevalence (Fig. 5). The geographical pattern of the malaria prevalence aggregated by councils and the heterogeneity among prevalence in schools within regions separated by survey phase is shown in Additional file 5: Fig. S5.

## Discussion

Almost 50,000 children from 537 public primary schools across all transmission zones in Tanzania were interviewed and tested for malaria infection. The nationwide malaria prevalence was 21.6%, marked by high variation across transmission zones, and administrative borders. The malaria prevalence in Tanzania was low in the “middle corridor”, ranging from Arusha and Kilimanjaro in the northeast to Njombe and Rukwa in the southwest of Tanzania, and high in the northwest (mainly Lake Zone) and in the southeast lowlands of Tanzania. The geographical pattern was similar to the previously described geographical pattern of malaria prevalence and vulnerability [1, 44, 45]. Compared to the TDHS-MIS 2015–2016, the national and regional malaria prevalence among school children was higher than the prevalence among children under the age of 5 years (21.6 vs 14%) [25]. Although the trend across the regions was similar, the especially high prevalence in the Lake Zone was surprising. One possible explanation could be the El Ninõ Southern Oscillation (ENSO) unstable climate conditions, which had caused ‘dramatic malaria outbreaks’ before [1].

### Bed net ownership and use

The percentage of reported bed net ownership in the children’s household was much higher than the reported percentages in the TDHS-MIS (89 vs 65%). However, the trend among regions was similar, with lower percentages in the middle part of Tanzania and higher percentages in regions in the Lake Zone and Southern Zone of Tanzania. The high reported bed net ownership among school children could likely be influenced by recall, reporting or interviewer bias, leading to overestimated values, although it was found that school children give reliable answers about bed net coverage in their community [21]. The percentage of children generally sleeping under a bed net was higher than the percentage for children under the age of 5 years, who slept under a bed net the previous night (69.9 vs 54.5%) [25]. This is different from previous findings that school children were less likely to sleep under a bed net than other age groups [46]. The disparity may be explained by the differences in the terms used, whereas “general” bed net use is much broader than bed net uses the previous night, leading to an overestimation of bed net use among school children. While on the other hand, bed net use among children under the age of 5 decreased since the previous MIS survey in 2011–2012 [24, 25] which is attributed to variations in distribution campaigns. Bed net use was higher in urban than in rural councils, which is similar to the findings in the TDHS-MIS. Moreover, bed net ownership and use were higher among children with higher educated parents, which is concordant with the TDHS-MIS findings [24, 25], assuming parental education and socio-economic status of the household to be the same indicator as with findings from a survey in Uganda [47].

### Malaria and its risk factors

The small increase in the malaria prevalence by age at all transmission zones, as well as the lack of significant association between age and malaria, might be surprising. Whilst the proportion of children with malaria slightly increased by age in all transmission zones, it would have been expected that younger children have a higher prevalence in high transmission areas and that older children would have a higher prevalence in low transmission zones [48]. A significant association between age and malaria was found in Ghana [49] and for a high transmission zone in Kenya [19, 50], whereas no association was found in a school survey in Ethiopia and Côte d’Ivoire [51]. However, the association between malaria and age highly depends on the transmission intensity among many other factors [48, 52]. The lack of association in the univariable model could be due to the fact that it was not distinguished between low and high transmission zones.

## Limitations

### Statistical analysis

Considering the variety of putative risk factors for malaria and bed net use, the regression models were not fully adjusted and in future analysis, it would be interesting to include risk factors such as distance to nearest health facility, distance to nearest water body, population density, housing conditions and/or socio-economic status. Also, the model included TSI and altitude, which were correlated but remained significant in the multivariable log-likelihood ratio test. Children, excluded in model I, adjusted for education and eco-zone had significantly higher reported bed net use and were on average younger than the children included in the model. One possible reason for the higher percentage of bed net use in the excluded children would be the school net distribution campaign in Southern Tanzania (Lindi, Mtwara, Ruvuma) in 2013 [53]. This would be supported by the reported higher percentages of obtained bed net through schools in Lindi and Mtwara region in the TDHS-MIS [25]. Further analysis could use stratification by region or transmission zone, geo-spatial analysis or geographically weighted logistic regression to assess the spatial correlation and its association with risk factors of malaria or bed net use.

### Malaria testing

Malaria rapid diagnostic tests were used to test children for malaria, which may have led to an overestimation in high transmission areas since the RDT were found to be limited in their ability to distinguish between “active and resolved infections” [54]. Also, although not further investigated in the present analysis, the number of positive-tested children may be biased by the proportion of children who had taken anti-malarial treatment recently before the day of testing. In future, this information could be of use for evaluation of the performance of malaria diagnosis and treatment at sub-regional level among school children. In low transmission areas, the use of RDTs may underestimate true prevalence, since very low parasitaemia, which lies below the detection level of the rapid diagnostic tests, were missed [54, 55]. It has been recommended to use molecular detection tools in low transmission areas [55]. This would enable school surveys to track down remaining human parasite reservoirs, which would contribute to an improved evaluation of the progress of malaria elimination in low transmission zones [56]. Moreover, it would be of interest to evaluate the use of schools in addition to health facilities for active case detection in the community, to identify remaining parasite reservoirs in low transmission zones and to identify hotspots within the catchment area of the schools.

### Validity of children interviews

Self-reported values are likely to differ from the truth, depending on interviewer and respondent characteristics, and face-to-face interviews are more likely to lead to answers influenced by social expectations [57, 58]. Since interviews were not standardized, interviewer bias might have varying influence on results. However, considering the large scale and sample size, it is less likely that this had an impact on the results at council level.

### Representativeness of sample

The data were analysed without adding sampling weights to account for the varying probability of selection at sub-council level. Nevertheless, with respect to representativeness, the study design included a population-weighted selection of council and stratum. In addition, the lack of available data at sub-council level to determine sampling weights would have provided limited additional accuracy to the results. Another limitation of the representativeness would be school absenteeism at day of the survey, drop out and enrolment rates in schools. According to a national educational survey conducted in 2013, enrolment rates were lowest in Kigoma, Katavi and Manyara regions (72.9, 73.6 and 79.8%, respectively), and otherwise mostly above 90% [30]. School absenteeism caused by malaria infection could have introduced a ‘healthy child effect’. This would lead to an underestimation of malaria prevalence, especially in low transmission areas, where children would be more likely to show symptoms and stay at home when infected [10, 11]. In Tanzania, there is not much known about how well school prevalence reflects community prevalence, although a study in Kenya found that data obtained in schools may reflect the community [10, 59]. Moreover, the use of school survey data for sentinel surveillance at community level could be validated in further research.

### Seasonality

The SMPS, as all cross-sectional surveys, captures malaria infection prevalence only at a certain time point and seasonal variations are inevitable. In the middle corridor, with low malaria prevalence, the survey was conducted during the end of wet season in most of the councils, while around the Lake Zone and in the southern parts of the country, with high malaria prevalence, the survey was conducted during the beginning of the short rains and dry seasons. The authors acknowledge that in Tanzania there is no quantifiable way to adequately and entirely adjust for differences in timing of the survey with expected prevalence rate, and this is a challenge even in most sophisticated mapping models or malaria prevalence. However, most school children (5–16 years), including those in this study, carry asymptomatic infections [16], which are less likely to be treated; thus, it has been documented that school children tend to harbour such infection for long periods, over 5 months [60, 61], potentially reducing the influence of seasonality on malaria infections in this age group. Future alternative sampling methods might include surveys at the beginning of every term, or more frequent surveys throughout the year, similar to the approach of rolling malaria indicator surveys.

### Advantages of SMPS compared to MIS

This SMPS, which is powered with adequate sample size to provide malaria prevalence estimates at council and sub-council levels, provides a complementary approach to malaria surveillance and parasitological monitoring in a short period of time alongside with other national representative surveys, such as Tanzania DHS and MIS. The nationwide school survey is the first survey in Tanzania describing the malaria risk among school children and the geographical trend. This is of importance for malaria control and elimination, as malaria infections in school children are often asymptomatic, contributing to malaria transmission in the community as ‘hidden’ parasite reservoirs [16]. The SMPS costs US$10 per test performed compared to MIS, which costs an average of US$410 per test performed. However, apart from malaria testing, MIS also captures household information such as coverage indicators including ownership and use of bed net, use of intermittent preventive treatment (Sulfadoxine Pyrimethamine) during pregnancy, and social and behaviour change communication [26].

## Conclusion

The observed overall malaria prevalence of 21.6% ranging from < 0.01 (Arusha, Manyara) to 53.6% (Geita) among regions indicates similar malaria heterogeneity and patterns as reported in the national surveys (TDHS/MIS). Findings of this survey are useful for updating the malaria epidemiological profile and for stratification of the malaria transmission by region, council and age groups, which is essential for guiding resource allocation and prioritizing future malaria interventions.

## Additional files

**Additional file 1: Fig. S1.** Flow chart of sampling design.

**Additional file 2: Fig. S2.** Duration of data collection in days per survey phase and district.

**Additional file 3: Fig. S3.** Age histogram.

**Additional file 4: Fig. S4.** Malaria prevalence per age by transmission zone.

**Additional file 5: Fig. S5.** **A** Map of the observed prevalence among schools aggregated per council, separated by survey phase. **B** Boxplot showing the distribution of observed prevalence among schools per region, sorted by amount of rainfall in the 3 months preceding the survey and separated by survey phase.

## Abbreviations

ALu: artemether–lumefantrine
HMIS: Health Management Information System
MIS: Malaria Indicator Surveys
RDT: malaria rapid diagnostic test
NMCP: National Malaria Control Programme
SMPS: School Malaria Parasitaemia Survey
TDHS: Tanzania Demographic and Health Surveys
TSI: temperature suitability index

## Footnotes

1 If not otherwise stated, the described results refer to the multivariable regression analysis model II. The results from the regression model I can be obtained from Table 4.
