# Supplementary figures and images for: Nationwide school malaria parasitaemia survey in public primary schools, the United Republic of Tanzania
Source: Malar J. 2018 Dec 5;17:452. doi: 10.1186/s12936-018-2601-1 (PMC6280377; doi:10.1186/s12936-018-2601-1)

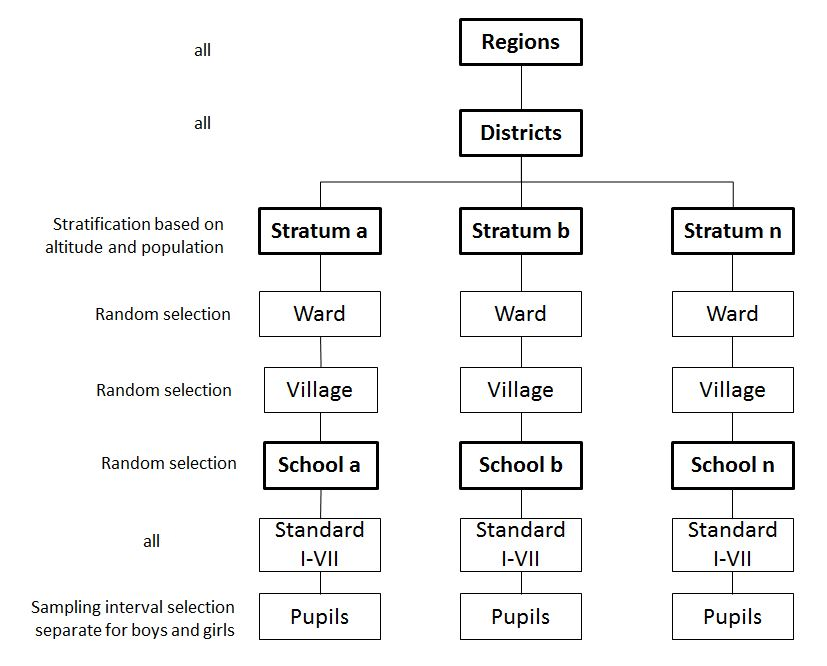

Supplement: Supplementary file 1 — Additional file 1: Fig. S1. Flow chart of sampling design. [file 12936_2018_2601_MOESM1_ESM.png]

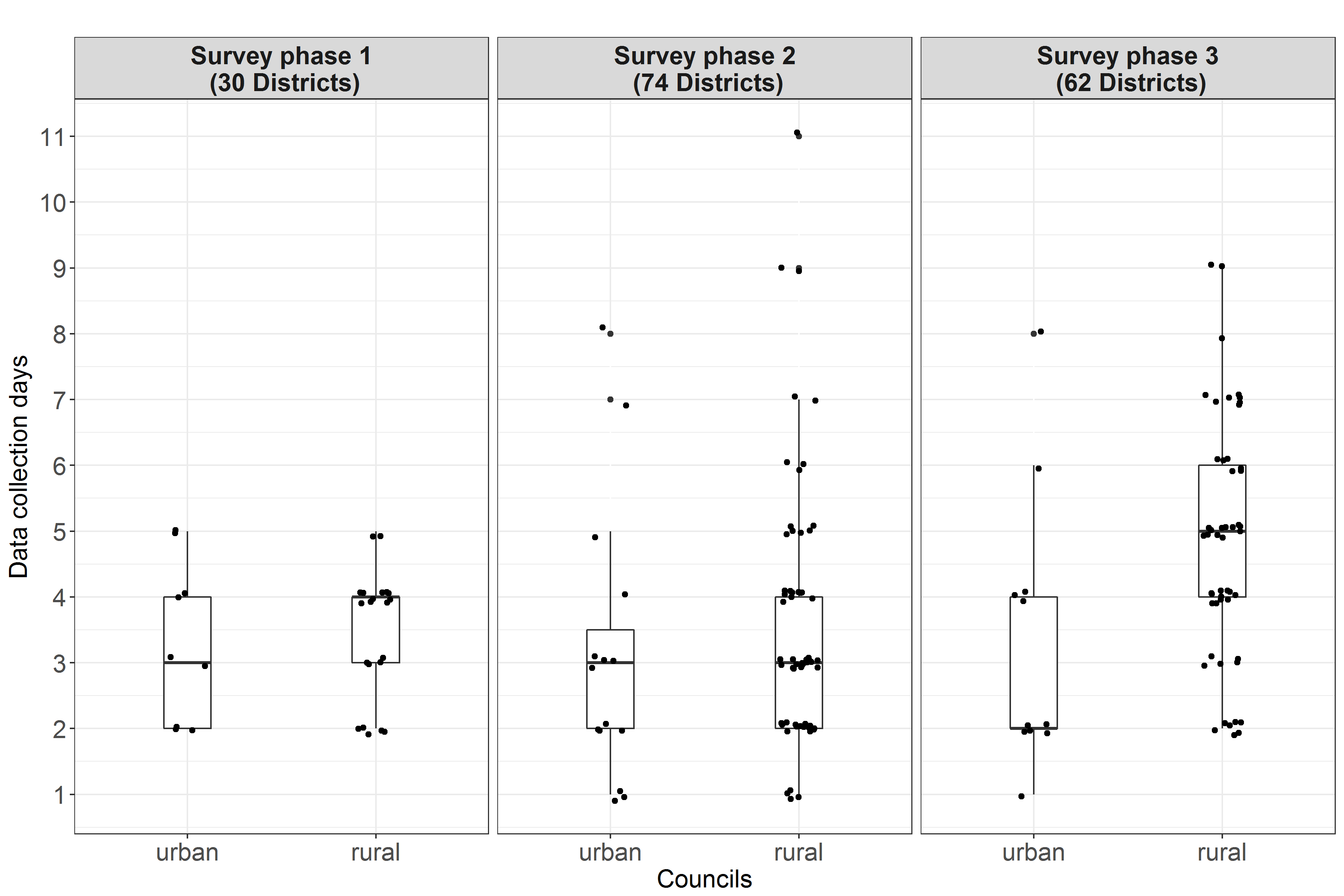

Supplement: Supplementary file 2 — Additional file 2: Fig. S2. Duration of data collection in days per survey phase and district. [file 12936_2018_2601_MOESM2_ESM.png]

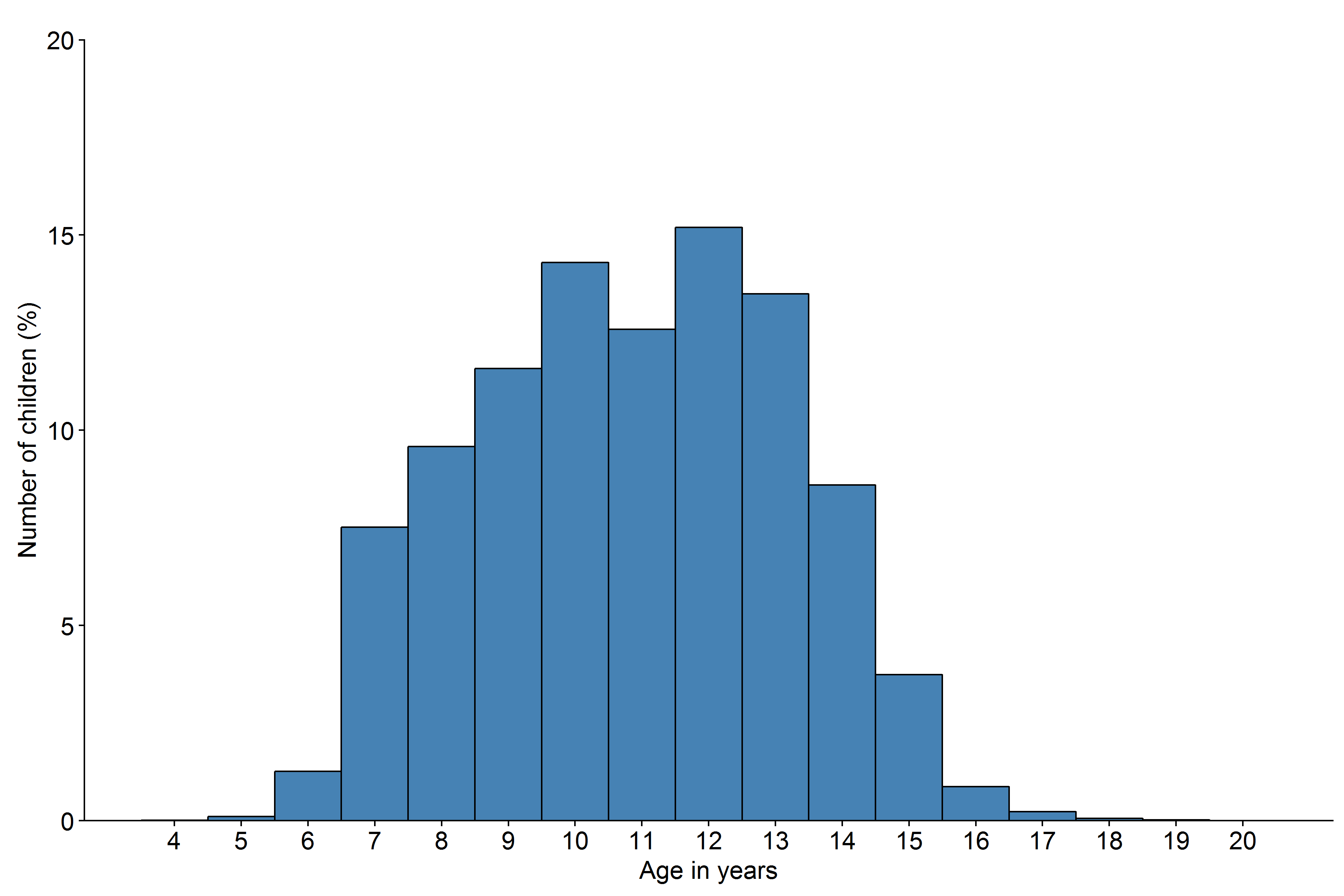

Supplement: Supplementary file 3 — Additional file 3: Fig. S3. Age histogram. [file 12936_2018_2601_MOESM3_ESM.png]

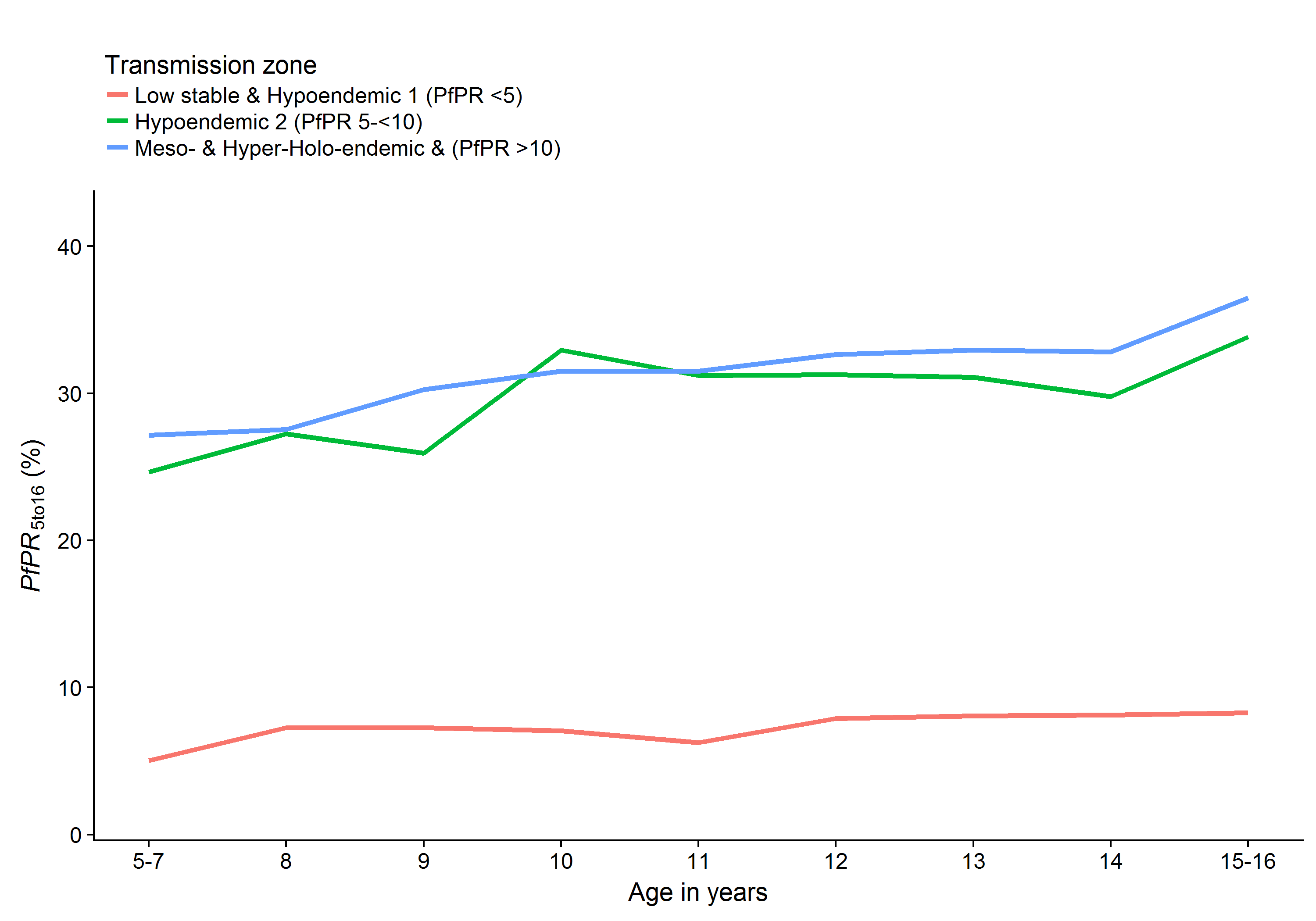

Supplement: Supplementary file 4 — Additional file 4: Fig. S4. Malaria prevalence per age by transmission zone. [file 12936_2018_2601_MOESM4_ESM.png]

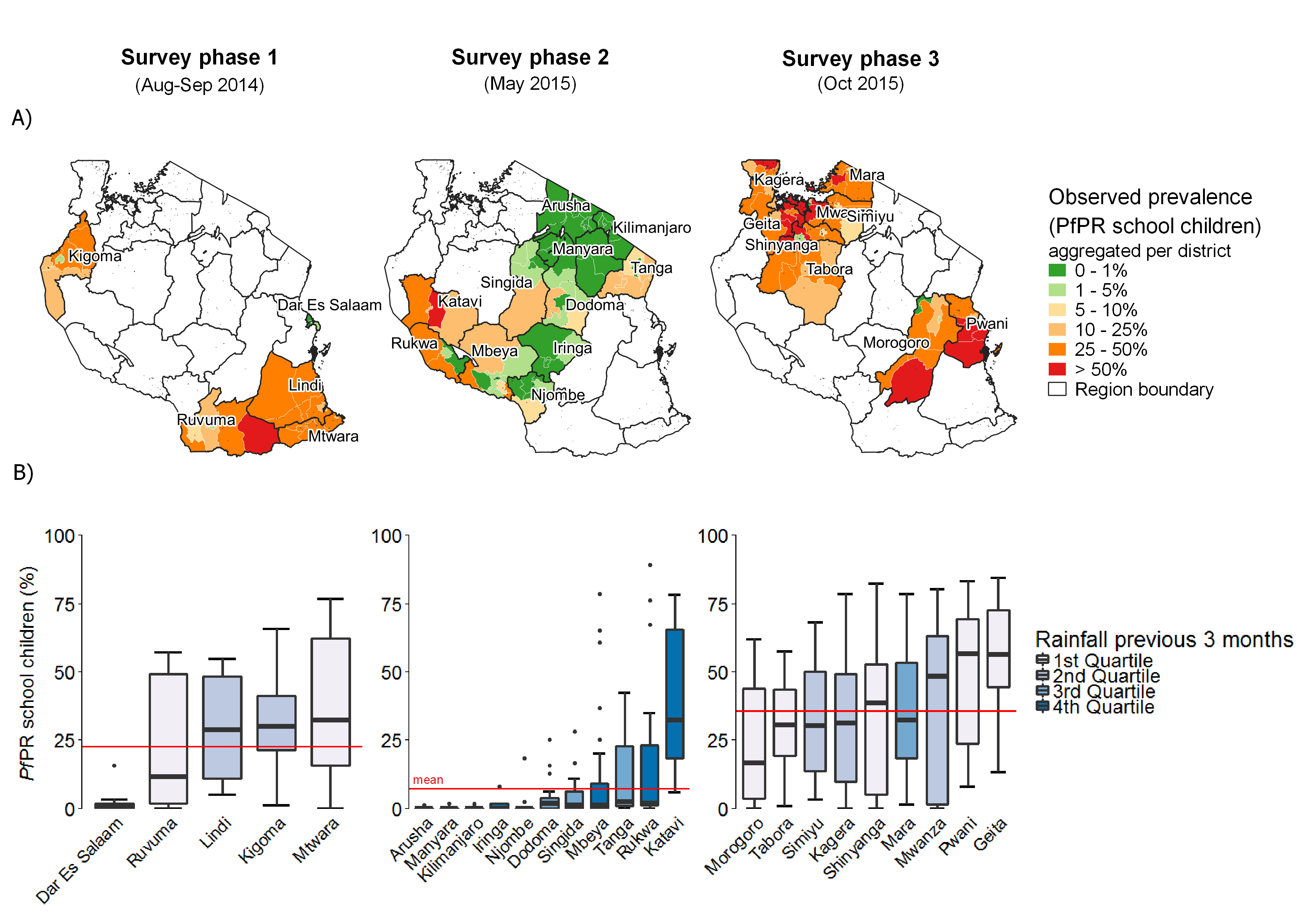

Supplement: Supplementary file 5 — Additional file 5: Fig. S5. A Map of the observed prevalence among schools aggregated per council, separated by survey phase. B Boxplot showing the distribution of observed prevalence among schools per region, sorted by amount of rainfall in the 3 months preceding the survey and separated by survey phase. [file 12936_2018_2601_MOESM5_ESM.png]
